# Older adults’ preferences for features of medication adherence technologies: a preference elicitation study

**DOI:** 10.1186/s44247-025-00222-z

**Published:** 2025-11-23

**Authors:** Ghada Elba, Bincy Baby, Ryan H. Griffin, Tejal Patel

**Affiliations:** 1 School of Pharmacy, University of Waterloo, Waterloo, ON Canada; 2 National Research Council Canada, Ottawa, ON Canada; 3 UW-Schlegel Research Institute of Aging, Waterloo, ON Canada

**Keywords:** Preference elicitation, Older adults, Adherence, Technologies

## Abstract

**Introduction:**

Older adults are at risk of medication non-adherence due to complex medication regimens and medication management challenges. Medication adherence technologies can help, but previous research demonstrates variability in usability and preferences of their features among older adults. Therefore, our objective is to examine older adults’ preferences for medication adherence technology features and their trade-offs to guide the development of these technologies. This will facilitate serving older adults better by addressing their needs and preference.

**Methods:**

Guided by the Patient-Centered Benefit-Risk Framework, we conducted a questionnaire based preference elicitation study where older adults ranked 10 medication adherence technology features identified through qualitative interviews, then identified acceptable trade-offs. Recruitment of our sample was based on convenience sampling, and the inclusion criteria was older adults above 60 years and older and able to speak and read English. The ranking was evaluated by calculating the relative importance using relative importance index (RII) and the trade-offs were assessed using win rate analysis. Statistical significance was assessed using Kruskal Wallis analysis.

**Results:**

Thirty older adults were recruited, of which twenty-three (mean age 73 years, 47.8% males) participated. The 10 reported features were button size, screen size, device size, compartment division, setting time and alarm, alarm sound, user-friendly leaflet, battery operated, locking features, and number of steps to set up the device. Screen size was ranked highest with relative importance index (RII) of 0.75. Win-rate analysis of trade-offs revealed that a user-friendly leaflet was the most frequently selected feature with p value < 0.001 (Kruskal Wallis test).

**Conclusion:**

This study highlights the importance of understanding the preferences of older adults to guide selecting the medication adherence technology that better meet their needs, as well as developing tools supporting medication management and adherence.

**Clinical trial number:**

Not applicable.

**Supplementary Information:**

The online version contains supplementary material available at 10.1186/s44247-025-00222-z.

## Introduction

Medication adherence, defined as taking medications as prescribed [1], is a key factor for effective disease management, improved clinical outcomes and mortality, especially for older adults who often manage multiple comorbidities [2]. Having comorbidities leads to the prescription of multiple medications at the same time [3], which increases the complexity of the medication regimens [4], and therefore adherence to the medications becomes challenging. Medication adherence technologies, such as devices or tools that assist in managing different medication regimens through organizing medication, sending reminders, remote monitoring and tracking adherence, are a potential solution for medication non-adherence [5, 6]. Several types of medication adherence technologies have been developed to assist older adults in managing their medications [7–9]. A scoping review that was conducted in 2021 identified 114 smart medication adherence technologies [8], while another study that was conducted in 2017 to systemically identify electronic medication adherence technologies available in Canada, identified 80 devices [10].

Since the effectiveness of using any device or tool depends on the functional attributes they offer and how they serve the objective of their intended use [11], the appropriate usability and acceptability of these attributes is critical for the effectiveness of use and is affected by how well they align with users’ preferences, needs, abilities, and challenges [12]. Understanding users’ preferences and capabilities as well as involving them in the design and development process of technologies are the core principles of human centered design and the study of human factors. According to the ISO 9241 − 210 standard [13], human centered design is defined as “an approach to systems design and development that aims to make interactive systems more usable by focusing on the use of the system and applying human factors/ergonomics and usability knowledge and techniques”, while human factors is defined as “the application of knowledge about human capabilities (physical, sensory, emotional, and intellectual) and limitations to the design and development of tools, devices, systems, environments, and organizations” [14].

Older adults, who are potential users of mediation adherence technologies, often experience many age-related changes including reduced physical ability, hearing loss, decreased vision and cognitive decline that can affect how they interact with technology and may result in variations in this interaction [15–17]. For example in a scoping review that identified barriers and facilitators to the use of e-health by older adults, interacting with small screens was a barrier for older adults with motor function decline and difficulties in learning new information related to the devices was also a barrier for people with memory challenges [18]. In this context, the study of human factors, especially in older adults, specifying their requirements, and applying them before and while designing new technologies will not only ensure the effective usability of and satisfaction with the device, but will also provide an appropriate solution for their challenges, ensure sustained use and create a safe and enjoyable experience [16, 19]. Several studies have highlighted that if the technology doesn’t align with older adults’ needs, they may not adopt the technology or they may personalise the technology in an unintended way, a process called bricolage [20, 21], which could potentially lead to frustrations and decrease safety while using it [22].

Older adults face specific challenges with technology and have different needs, priorities, confidence, attitudes and preferences with their use of technology [23]. To address these differences, when designing technology specifically for them, human centred design principles emphasis the importance of examining their preferences [24, 25]. Patient preference is defined as “relative desirability or acceptability to patients of specified alternatives or choices among outcomes or other attributes that differ among alternative health interventions,” [26]. Actively engaging older adults and addressing their preferences has proven beneficial [27–31]. Ethically, patient engagement promotes autonomy, an important need for older adults. It also helps improve patients’ knowledge, align expectations, and reduce decision conflicts [27]. Addressing patients’ preferences in medication-related decisions improves patient satisfaction and medication adherence [28, 29]. Furthermore, accommodating patients’ preferences is also associated with increased motivation and better outcomes [31]. Finally, considering patients’ preferences helps design and select appropriate services that meet patients’ needs, promoting better personalization of care [30].

When choosing between different medication adherence technologies, older adults might navigate multiple features, for which they may have varying preferences [6, 32, 33]. For example, some older adults may prefer simplicity and ease of use, while others may favour alarms and large button sizes [32, 33]. Some older adults may prioritize portability for convenience while travelling, while others prefer compact devices to save space or to hide away for privacy [33]. Given these variations in older adults’ preferences, it is imperative to understand their preferences and ensure that the technologies meet their needs and preferences.

While some studies have reviewed the available medication adherence-supporting technologies [7, 34] and others have explored their usability, acceptability, workload, and effectiveness [35–38], systematically examining and quantifying the specific features that users consider important while managing their medications remains under-investigated. For example, a study by Russell et al. [39] highlighted some of the preferred features like medication lists and consolidated schedules, reminder alerts and recording time of taking medication. However, it did not systematically measure the relative importance of these features when compared to others nor the trade-offs, which is deciding between two features when both can not be incorporated in one product, between these functionalities’ users were willing to make. Moreover, the studies that examined user feedback and acceptability of medication supporting technologies [33, 38, 40] did not apply preference elicitation methods, like rating, ranking, discrete choice experiments or best-worst scaling to understand the weight and value of the technology features. Finally, research on tools that support medication adherence does not address the unique preferences and needs of older adults who may have varying levels of technology literacy as well as age related changes that influence their preferences. As most technologies offer both features that are both preferred and needed and those that are not, it is important to gauge the trade-offs older adults are willing to make when choosing their devices.

Therefore, in this study, we aim to fill this gap by systematically examining the preferences for features of medication adherence technologies amongst older adults by measuring the relative importance of the medication adherence technology features and examining the trade-offs. In this study, we focused on standalone, electronic and smart medication adherence technologies, such as automated pill dispensers and electronic medication organizers, rather than mobile phone apps.

## Methods

### Study design

This study was designed as a 2-stage interview-based study that used a ranking method and pairwise comparison for preference elicitation. In this study, the Patient Centered Benefit Risk (PCBR) framework [41]guided our methodology. The PCBR framework was developed by the medical device innovation consortium (MDIC) to incorporate patients’ preferences and values in the benefit/risk assessment of different medical devices. According to the PCBR framework [41], data collected include: (a) the list of attributes of the tool investigated, (b) the relative importance of these attributes to the users, and finally, (c) the trade-off the users are willing to make between these attributes (see Appendix). The list of attributes can include features of technology, outcomes of a treatment plan, health states or their probabilities. Attributes can be identified with qualitative methods, literature reviews and experts’ opinions. The relative importance metric is a higher level of information that is particularly concerned with giving each attribute a weight to be compared to the other attributes. Four methods are commonly used for assessing relative importance [41] including structured weighting, stated preferences, health utility and revealed preferences. For this study, structured weighting techniques were used, as they are useful for multilevel attributes, and they include methods like ranking, swing weighting, and point allocation.

Finally, according to the PCBR, trade-offs offer the highest level of information about which attributes the participants are willing to trade for others by comparing them to the other attributes or changing the levels of attributes.

### Identifying the list of attributes

Preferences were investigated for the attributes listed below:

- Screen size*.
- Button size*.
- Device size*.
- Compartment division*.
- Setting time and alarm*.
- User friendly leaflet.
- Having an alarm sound*.
- Locking features*.
- Battery operated*.
- Number of steps to set up the device.

*These attributes pertain to the hardware component of the devices.

Selection of these attributes was determined through an interview-based study among participants who tested different medication adherence technologies during a user experience study [32]. In this user experience study, older adults tested the usability of thirteen different medication adherence technologies, with each participant testing up to four different devices [32]. As a component of the usability study, participants were invited to a semi-structured interview where they were asked about the preferred and most useful features as well as the worst and the least useful features of the medication adherence technology they tested. A list of preferred features from this analysis was used to identify the ten most reported features to include in the relative importance and the trade-off exercise. The primary reason for selecting only ten features was to decrease the cognitive load and risk for overwhelming our older adult participants and minimize the confusion and frustration associated with such activities. We conducted two preference elicitation tasks: a ranking exercise to measure the relative importance of the attributes (stage 1) and a pairwise comparison exercise (stage 2) to measure the trade-offs between those attributes.

### Measuring relative importance

In the ranking exercise, the participants were given the set of attributes and asked to rank them from 1 to 10 according to their relative importance, where 1 is the most important and 10 is the least important. This activity quantifies the preferences by measuring how much each attribute matters to the participant compared to the other reported attributes.

### Measuring trade-offs

In this task, participants were given different sets of 2 attributes and asked to choose the more important one to identify which feature they were willing to trade-off. We created all the possible combinations of attributes from the 10 attributes previously identified to ensure comprehensive assessment.

### Study population

Participants were included if they were 60 years or older, able to speak and communicate in English, and willing to provide informed consent. Individuals who were unable to speak or read English were excluded.

### Ethics approval

This study received approval from the University of Waterloo Research Ethics Board (REB #45203).

### Consent to participate

All participants provided informed consent prior to data collection, and confidentiality was maintained throughout the study by anonymizing all personal data.

### Data analysis

For the ranking analysis, the rank median of each attribute was calculated, and the relative importance was calculated by the Relative Importance Index (RII) [42]. The RII was calculated using the following equation:

$$\:RII=\:\frac{\sum\:W}{A\times\:N}$$

Where W is weights assigned by participants to each attribute, A is the highest weight and N is the total number of participants.

For the trade-offs analysis, we calculated the win rate [43], which is the number of times a certain attribute was preferred over another in the pairwise comparison. For example, if an attribute appeared in 10 pairwise comparisons and was selected 7 times, this would equate to a win rate of 70%. Kruskal Wallis test was used to determine the statistical significance of the results of the win rate analysis.

$${\mathbf{Win}}{\text{ }}{\mathbf{rate}} = \left( {\frac{\begin{gathered} number\:of\:times\:an\:attribute\:was\:selected \hfill \\ in\:the\:pair\:wise\:comaprison\:sets \hfill \\ \end{gathered} }{\begin{gathered} number\:of\:times\:the\:attribute\:appeared \hfill \\ in\:all\:the\:pari\:wise\:comparison\:sets \hfill \\ \end{gathered} }} \right) \times 100$$

## Results

### Demographics

Of the 30 participants invited, 23 agreed to participate in the study. The mean age of the participants was 73 ± 5.7 years (mean ± standard deviation). Twelve (52.1%) of the participants were female. Each participant tested three to four devices in the usability study. Demographics are displayed in Table 1 below.

**Table 1:** Demographics

Age (mean ± standard deviation)	73 ± 5.7
Sex (%)	
Male Female	47.8 52.1
Number of medications they take (mean ± standard deviation)	3.7 ± 2.5
Level of education (%)	
Masters/Doctoral/Professional Bachelors Non university diploma Trade certificate/diploma High school Below high school	34.8 21.8 17.4 4.3 17.4 4.3
Previous use of medication adherence technology (%)	52.1

### Ranking exercise and relative importance analysis

Of our sample of 23 participants, 22 completed the ranking exercise. The participants ranked the screen size and button size as the most important features in the technologies, with a median rank of 3. Whereas the availability of a locking feature was ranked lowest with a median rank of 9. These results are demonstrated in Table 2.

**Table 2:** Median rank of all attributes

Attribute	Median rank on a scale of 1–10 with 1 as most and 10 as least important	Interquartile range (IQR)
Screen size	3	1–5
Button size	3	2–5
Device size	5	3–7
Compartment division	4.5	2–8
Setting time and alarm	6	4–7
User friendly leaflet	4	3–6
Alarm sound	6.5	5–9
Locking features	9	7–10
Battery operated	8.5	6–10
Number of steps to set up the device	6.5	4–9

Table 3 represents the RII scores, which indicate how important each attribute is to the participant when compared to the other attributes. Higher scores indicate greater importance. The results indicate that the screen size had the highest Relative Importance Index score (0.75) while the number of steps to set up a device was lowest at 0.45.

**Table 3:** Relative importance among all participants

Attribute	RII
Screen size	0.75
Button size	0.74
Device size	0.60
Compartment division	0.58
Setting time and alarm	0.52
User friendly leaflet	0.67
Alarm sound	0.43
Locking features	0.25
Battery operated	0.32
Number of steps to set up the device	0.45

### Trade-off analysis

The trade-off exercise was also completed by 22 out of 23 participants (see Table 4). The majority of participants (82–91%) were willing to trade-off having an alarm sound, a locking feature for a larger button size, compartment division, user-friendly leaflet, and a small number of steps to set up the device. Moreover, 82% of the participants are willing to trade-off the button size and having a battery-operated device to have a user-friendly leaflet and a small number of steps to set up the device.

**Table 4:** Choice matrix for trade-off analysis (*n* = 22)

	Device size	Button size	Screen size	Compartment division	Setting time and alarm	User friendly leaflet	Alarm sound	Locking features	Battery operated	Number of steps to set up device
Device size										
Button size	14									
Screen size	17	12								
Compartment division	14	12	9							
Setting time and alarm	15	12	9	10						
User friendly leaflet	15	18	13	12	9					
Alarm sound	6	3	5	4	6	4				
Locking features	5	3	5	4	5	4	8			
Battery operated	8	6	6	8	8	6	13	12		
Number of steps to set up device	15	12	8	15	7	9	20	18	18	

The number in the table corresponds to the number of participants who selected the feature in the left column when compared to the feature in the first row

The trade-off analysis, as determined by the win rate, is presented in Table 5. The screen size and the user-friendly leaflet were the most selected attributes, and the availability of a locking feature was the lowest attribute chosen (*p *< 0.001) .These results were statistically significant.

**Table 5:** Win rate analysis for all attributes among all participants

Attribute	Mean number of times an attribute was selected in the pairwise comparison sets (± SD )	Win rate Percentage (%)	*P* value*
User friendly leaflet	6.0 **±** 2.8	67	< *p* < 0.001
Screen size	5.8 ± 2.5	64	
Number of steps to set up the device	5.5 ± 2.3	61	
Setting time and alarm	5.5 ± 2.3	61	
Compartment division	5.2 ± 2.0	58	
Button size	5.1 ± 2.0	57	
Device size	4.0 ± 2.0	44	
Battery operated	3.2 ± 2.9	36	
Alarm sound	2.4 ± 1.9	27	
Locking features	2.2 ± 1.9	24	

*Kruskal Wallis test

## Discussion

This study was designed to explore the preferences of older adults for the attributes of different medication adherence technologies. Exploring preferences of older adults, our target population, is an essential aspect of human-centered design, as it allow us to understand their needs and determine the design solutions needed based on their preferences. To the best of our knowledge, this is the first study to systematically assess the preferences of older adults for the features of medication adherence technologies, conducted through a preference elicitation study.

This study determined that the screen size and button size were ranked as the most important attributes by older adult participants, with screen size having a slightly higher RII. This is consistent with the results reported by Yu et al. [44]. In this study, Yu et al. investigated the effect of different features of buttons including button size on user performance in older adults and younger adults with a smart home interface prototype; 80% of the older adults preferred button sizes more than 20 mm in size. Additionally, older adults demonstrated shorter task completion time when using 20 mm buttons compared to smaller button sizes with a significant difference (*p* < 0.001) [44]. Moreover, participants with self-reported conditions such as multiple sclerosis, cerebral palsy, parkinson’s disease, Huntington’s disease, or tremors preferred a bigger button size, 20 mm or more, due to physical barriers. They also concluded that the increase in button size continued to yield better performance scores, as noted by the percentage of average misses (30% decrease between 25 mm and 30 mm, *p* < 0.001), percentage of average errors (22% decrease in errors between 20 mm and 25 mm, and 15% decrease between 25 mm and 30 mm, *p* < 0.05) and average task completion time (9.6% decrease as button size increased from 10 to 15 mm, *p* < 0.001). This was among those individuals with maximum improvement in performance with button sizes 30 mm when performance started to plateau, compared to healthy individuals whose performance did not significantly increase with increasing button size [45].

The screen size had a relatively important attribute to the participants with an RII of 0.75. This is supported by Zhou et al., where they investigated the effect of screen size and input methods on older adults’ performance, preference, and acceptance [46]. They concluded that older adults preferred larger displays (7 in. 1.5 times more than 5 in.) and found larger screens (7 in. compared to 5 in.) was easier to use especially in typing tasks. This could be attributed to that an appropriate screen size, especially a bigger size, can provide a wider view field and a richer and closer-to-reality experience [46]. In addition, having a bigger screen was associated with better control due to having a larger area for interaction [46, 47] and accommodation for accidentally pressing two buttons due to limited space between buttons, as well as better readability [47].

The average win rate was significantly different between the different features with the highest for having a user-friendly leaflet (*p* < 0.001). Leaflets serve as an important source of information for many older adults for several reasons; firstly, they are a familiar method for acquiring information [48]. Many older adults take multiple medications, where medication leaflets may provide a default way to gain knowledge about their medication regimen [49]. Similarly, they will access a leaflet or instruction manual for information about devices. Moreover, leaflets written in plain language without the use of medical or technical terminology, are useful for facilitating the understanding of the operation of complex tools [49]. Being paper based is another factor that motivates older adults to use leaflets, a finding reported by Hammar et al. [48]. Older adults prefer to use paper-based information documents compared to online digital resources due to limited accessibility to these resources, uncertainty about the right or reliable sources, and usability or functionality issues [48, 50]. Furthermore, Hammer et al. [48], found significant differences between older adults and younger individuals in terms of requesting leaflets, with older adults requesting these if not included in the packaging (*p* < 0.05). Finally, many older adults prefer to read leaflets for new devices to learn how to use it by self-training and trial and error [51]. Therefore, having a user-friendly and an easy-to-follow instruction leaflet is preferred by older adults to ensure they can learn to use the new technology, which may impact the adoption of new technologies. By systematically evaluating the preferences and giving them weights, these findings could provide guidance for developers who want to design medication adherence technologies based on the preferences of their users.

### Strengths and limitations of the study

This study has several strengths. First, we were able to systematically measure older adults’ preferences for some key features of medication adherence technology. The combination of both a ranking exercise and the trade-off analysis provided comprehensive understanding of the relative importance of these features to older adults and the features they are willing to trade-off. However, the study also has some limitations, for example, having a small sample size would limit the generalizability of the results as the sample may not be representative of the whole population. Furthermore, although a previous classification study conducted by this research team notes many more features, this study was conducted with a selection of 10 features to reduce the burden on older adults during the study, this approach may not capture all features that matter to older adults and require an examination of preferences and trade-offs. Moreover, as older adults are a heterogenous group with varying preferences and abilities, our study did not recruit an adequate sample that enables a comparison of the preferences of individuals with different age-related functional abilities. 

## Conclusion

This study highlighted the preferences of older adults for the features of medication adherence technology. The findings showed that screen size, button size and user-friendly leaflets are important features for older adults, while having a locking feature is less important. Therefore, it is highly imperative to understand the preferences of the targeted population to address their needs and ensure adoption, acceptability, and usability of these technologies. In addition, understanding the preferences for older adults with different age-related barriers will ensure the design of technologies that accommodate their challenges and, therefore, optimize satisfaction and product adoption.

### Future directions

Based on these findings, future studies should be directed towards expanding the sample to improve generalizability and subgroup analysis. Moreover, having in-depth interviews as an additional stage would allow us to inductively elicit the reasons behind the preferences for certain features and to further understand why they are prioritizing some features compared to others. This step is beneficial especially with older adults who have different age-related barriers, demographics, comorbidities, medication regimens, technology literacy, and socioeconomic status, all of which would influence their preferences for features. Additionally, investigating additional user-driven features (more that the 10 we investigated) and what older adults would like to have when designing new devices is essential to ensure comprehensive results. This can also be better addressed through co-design methods. Furthermore, recruitment should target individuals with a single barrier and individuals with multiple barriers to allow for comparison. Finally, an appropriate statistical test should be considered to detect the influence of different barrier combinations on preferences.

## Supplementary Information

Below is the link to the electronic supplementary material.

Supplementary Material 1

## Data Availability

Data are available upon request to the corresponding author.

## Abbreviations

RII: Relative Importance Index
PCBR: Patient Centered Benefit Risk
MDIC: Medical Device Innovation Consortium
IQR: Interquartile Range
SD: Standard Deviation
