# Supplementary material for: Older adults’ preferences for features of medication adherence technologies: a preference elicitation study
Source: BMC Digit Health. 2025 Nov 23;3(1):86. doi: 10.1186/s44247-025-00222-z (PMC12641034; doi:10.1186/s44247-025-00222-z)
Supplement: Supplementary file 1 — Supplementary Material 1 [file 44247_2025_222_MOESM1_ESM.docx]

Appendix

Questionnaire guide

These are a set of features of the devices we have tested today, can you please rate the importance of each feature on a scale of 1 to 10 and then rank them from 1 to 10 where 1 is the most important and 10 is the least important?

| **Rank** | **Feature** |
| --- | --- |
|  | Screen size |
|  | Button size |
|  | Device size |
|  | Compartment division |
|  | Setting time and alarm |
|  | User friendly leaflet |
|  | Alarm sound |
|  | Locking features |
|  | Battery operated |
|  | Number of steps to set up the device |

Which one do you prefer from these two options

| **Option A** | **Option B** | **Your choice** |
| --- | --- | --- |
| Device size | Button Size |  |
| Device size | Screen Size |  |
| Device size | Compartment Division |  |
| Device size | ease of setting time and alarm |  |
| Device size | user friendly leaflet |  |
| Device size | alarm sound |  |
| Device size | locking features |  |
| Device size | battery operated |  |
| Device size | number of steps to setup the device |  |
| Button Size | Screen Size |  |
| Button Size | Compartment Division |  |
| Button Size | ease of setting time and alarm |  |
| Button Size | user friendly leaflet |  |
| Button Size | alarm sound |  |
| Button Size | locking features |  |
| Button Size | battery operated |  |
| Button Size | number of steps to setup the device |  |
| Screen Size | Compartment Division |  |
| Screen Size | ease of setting time and alarm |  |
| Screen Size | user friendly leaflet |  |
| Screen Size | alarm sound |  |
| Screen Size | locking features |  |
| Screen Size | battery operated |  |
| Screen Size | number of steps to setup the device |  |
| Compartment Division | ease of setting time and alarm |  |
| Compartment Division | user friendly leaflet |  |
| Compartment Division | alarm sound |  |
| Compartment Division | locking features |  |
| Compartment Division | battery operated |  |
| Compartment Division | number of steps to setup the device |  |
| ease of setting time and alarm | user friendly leaflet |  |
| ease of setting time and alarm | alarm sound |  |
| ease of setting time and alarm | locking features |  |
| ease of setting time and alarm | battery operated |  |
| ease of setting time and alarm | number of steps to setup the device |  |
| user friendly leaflet | alarm sound |  |
| user friendly leaflet | locking features |  |
| user friendly leaflet | battery operated |  |
| user friendly leaflet | number of steps to setup the device |  |
| alarm sound | locking features |  |
| alarm sound | battery operated |  |
| alarm sound | number of steps to setup the device |  |
| locking features | battery operated |  |
| locking features | number of steps to setup the device |  |
| battery operated | number of steps to setup the device |  |
